# A Rare Case of Coexisting 22q11.2 Deletion Syndrome and Cornelia De Lange Syndrome: A Case Report and Review of the Literature

**DOI:** 10.7759/cureus.96366

**Published:** 2025-11-08

**Authors:** Emily George, Katina Kontos

**Affiliations:** 1 Paediatrics, Wrightington, Wigan and Leigh Teaching Hospitals NHS Foundation Trust, Greater Manchester, GBR; 2 Community Paediatrics, University Hospital Llandough, Cardiff, GBR

**Keywords:** 22q11.2 deletion syndrome, co-existing genetic syndromes, cornelia de lange syndrome, digeorge syndrome, genetic diagnosis, multidisciplinary care

## Abstract

The coexistence of multiple genetic syndromes in a single individual is rare but clinically significant, as it can lead to delays in diagnosis and management. To ensure high-quality, patient-centred care, clinicians must remain alert to the possibility of dual diagnoses. This case report describes a rare presentation encountered in a clinical genetics setting and highlights its key learning points, complemented by a review of the relevant literature. We report the case of a 15-year-old girl born prematurely with a complex medical history including developmental delay, feeding difficulties, joint hypermobility, vacant episodes, and endocrine disturbances. At age three, she received a diagnosis of 22q11.2 deletion syndrome. However, there remained concerns that this diagnosis did not fully account for her severe neurodevelopmental and feeding difficulties. As such, at age 14, a decade later, she was enrolled in the Deciphering Developmental Disorders (DDD) study, which identified a pathogenic variant in SMC3, confirming an additional diagnosis of Cornelia de Lange syndrome. This case highlights the challenges associated with coexisting diagnoses, illustrated by the 10-year interval between this patient’s first and second diagnoses. Confirmation of the second additional genetic diagnosis clarified previously unexplained features. This clarification enabled targeted multidisciplinary care and informed discussions with the patient and family regarding prognosis and management. With advances in genomic knowledge and improved access to genetic testing, cases of coexisting genetic syndromes are likely to become increasingly common. Recognising when a patient’s presentation is not fully explained by a single syndrome is critical. Identifying coexisting genetic conditions enables a comprehensive approach to surveillance, follow-up, and genetic counselling for the patient and wider family.

## Introduction

The coexistence of multiple genetic conditions in an individual is rare but clinically important, with significant implications for both physical and psychological well-being [1]. Early recognition of features suggestive of a genetic diagnosis, such as dysmorphic features, congenital anomalies, or developmental concerns, can prompt appropriate investigation and intervention.

While clinicians typically seek a single, unifying diagnosis, there are cases where clinical features remain unexplained by an initial diagnosis. Failure to consider coexisting diagnoses risks delayed diagnosis and management, highlighting the need for clinical open-mindedness and an individualised approach to patient care.

The increasing use of genomic testing has greatly improved the ability to identify rare diseases. Recent genomic studies have shown that between 2% and 7% of individuals with developmental disorders possess more than one pathogenic variant - genetic changes known to contribute to disease - contributing to their phenotype [2]. Studies, including the Deciphering Developmental Disorders (DDD) study, have reinforced that dual diagnoses are becoming increasingly recognised as genomic technologies expand [3-4].

DiGeorge syndrome, also known as 22q11.2 deletion syndrome, and Cornelia de Lange syndrome are well-recognised genetic disorders with separate aetiologies, characteristic features, and associated complications [5-6]. 22q11.2 syndrome, affecting approximately one in 4,000 individuals, results from a microdeletion - a small loss of genetic material - at chromosome 22q11.2 and typically presents with congenital heart defects, immunodeficiency, palatal abnormalities, developmental delay, and characteristic facial features [5-7]. Cornelia de Lange syndrome is less common (one in 10,000-30,000) and results from pathogenic variants in a number of genes, including NIPBL and SMC3. It is characterised by growth restriction, limb anomalies, feeding difficulties, developmental delay, and distinctive facial features [6-8]. While there is some overlap in clinical features, namely, developmental delay and behavioural difficulties, their aetiologies and other clinical manifestations are distinct [5-6].

Given the increasing recognition of dual genetic diagnoses in genomic studies, reporting rare cases of coexisting syndromes is valuable to inform clinical practice. To our knowledge, having searched the current literature, their coexistence has not previously been reported. We present the case of a paediatric patient diagnosed with 22q11.2 syndrome and later Cornelia de Lange syndrome, offering insights into her journey through care, highlighting the diagnostic challenges associated with dual diagnoses, and reflecting on the key learning points. In doing so, we review the literature relevant to their coexistence.

## Case presentation

A female infant was born prematurely at 27+6 weeks' gestation following an uncomplicated pregnancy and spontaneous vaginal delivery. She required resuscitation at birth and was admitted to the Special Care Baby Unit, where she remained an inpatient for four months. Neonatal complications included respiratory distress syndrome and feed-associated apnoea, requiring respiratory support. At birth, she was on the 9th centile for weight and between the 2nd and 9th centiles for height, placing her below average for size. A soft systolic murmur was noted during the neonatal period, and an echocardiogram demonstrated a patent ductus arteriosus that later resolved spontaneously. She was also found to have an aberrant right subclavian artery, with no haemodynamic significance. 

During the neonatal period, subtle dysmorphic features were noted, including upward-slanting palpebral fissures, low-set ears, and small hands and feet. In view of these findings, in combination with her significant feeding difficulties, she was referred to the clinical genetics team and enrolled in the Deciphering Developmental Disorders (DDD) study. 

These feeding difficulties persisted throughout childhood, accompanied by excessive oral secretions, for which she underwent botulinum toxin injections to the salivary glands and a pharyngoplasty. Initial anti-reflux therapies provided little benefit. Following assessment by speech and language therapy and ENT, the child was fed via nasogastric tube and subsequently via percutaneous endoscopic gastrostomy (PEG), which remains in place today. Nutritional support resulted in satisfactory catch-up growth, with her weight centile increasing from the 9th at birth to the 50th by age 14. Her height trajectory remained low, tracking along the 3rd centile at age 14.

Physical examination demonstrated hypermobility, small, high-arched feet, and progressive scoliosis. The patient also had patella and bilateral congenital radial head dislocations. She initially suffered frequent lower respiratory tract infections in the first three years of life - requiring approximately 12 courses of antibiotics per year - but this markedly improved once she commenced on prophylactic antibiotics. She experienced recurrent headaches and a few (less than 10) vacant episodes; therefore, magnetic resonance imaging (MRI) brain and an electroencephalogram (EEG) were arranged, and both were generally unremarkable, so she was discharged from neurology. Endocrine complications included autoimmune hypothyroidism and intermittent hypoparathyroidism, while ophthalmological review revealed hypermetropia. She exhibited global developmental delay and intellectual disability, requiring enrolment in a special school, and displayed autistic features with associated anxiety.

At age three and a half years (May 2013), comparative genomic hybridisation (CGH) analysis confirmed 22q11.2 deletion syndrome. However, her profound neurodevelopmental impairment and severe feeding difficulties were not consistent with this diagnosis. Analysis through the DDD study subsequently identified a pathogenic heterozygous SMC3 variant, establishing a concurrent diagnosis of Cornelia de Lange syndrome, over a decade later, in April 2024, consistent with the previously unexplained features. Table 1 provides a chronological summary of relevant clinical events.

**Table 1:** Chronological overview of key clinical events.

Date	Clinical event
Nov 9	The patient was born with respiratory distress syndrome and feed-associated apnoea requiring respiratory support, an NG tube inserted, diagnosed with patent ductus arteriosus and an aberrant right subclavian artery, and dysmorphic features were noted.
Mar 10	Discharged from the neonatal unit with an NG tube in situ
May 11	PEG inserted
May 13	Diagnosed with 22q11.2 deletion syndrome
May 14	Diagnosed with hypothyroidism
Jun 16	Diagnosed with patella and radial head dislocations
Sep 18	Diagnosed with hypoparathyroidism
Apr 24	Diagnosed with Cornelia de Lange syndrome

**Figure 1:**
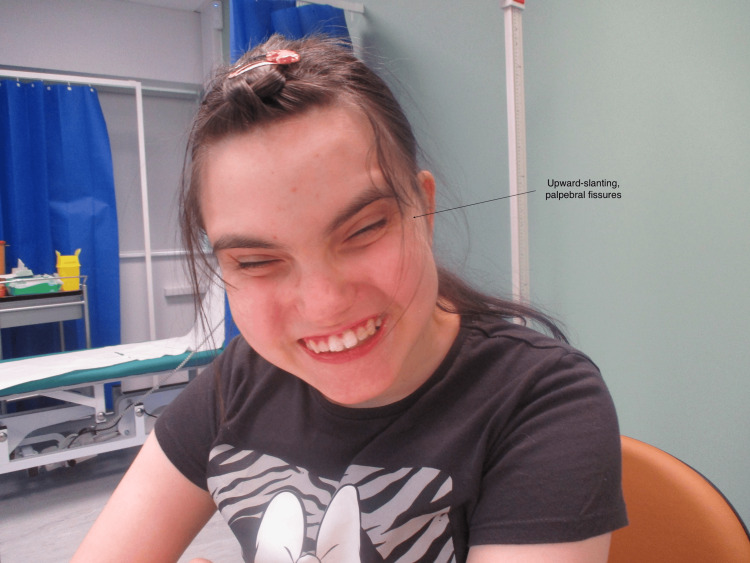
Recent clinical photograph of the 15-year-old patient, showing characteristic feature - upward-slanting palpebral fissures.

Currently, this patient is well and remains under the care of several multidisciplinary teams. She continues to be PEG-fed and remains in a supported learning environment, undergoing formal neurodevelopment assessment. On examination in the present day, she demonstrates dysmorphic features, including upward-slanting palpebral fissures and low-set ears (Figure 1).

Signed, informed consent to publish this image in an online journal was received from the parent of the patient. 

## Discussion

This case describes the rare coexistence of 22q11.2 deletion syndrome and Cornelia de Lange syndrome - two genetically distinct conditions with overlapping clinical features.

Medical teaching generally encourages clinicians to apply Occam’s razor, a principle that favours the simplest explanation, namely, a single unifying diagnosis that accounts for all clinical findings. However, Hickam’s dictum reminds us to consider multiple, concurrent diseases in patients with complex or atypical presentations. This case is an example of the latter [9].

This patient was initially diagnosed with 22q11.2 deletion syndrome, a condition affecting around one in 4,000 individuals [5]. It most commonly results from a microdeletion at chromosome 22q11.2 and arises *de novo* in around 90% of cases, being inherited in an autosomal dominant manner in the remainder [5-7]. The clinical spectrum is variable, but classical features include congenital heart defects, immunodeficiency (due to thymic hypoplasia), palatal abnormalities, and developmental delay/intellectual disability and psychiatric disorders, including schizophrenia [5-7]. Characteristic facial features may also be present, including a broad nasal bridge, a small chin, and hooded eyelids. Less common manifestations may include hypocalcaemia (due to hypoparathyroidism), renal anomalies, and behavioural or feeding difficulties [5-7]. The severity can range from mild features to severe multi-system involvement. Diagnosis is established through molecular genetic testing, with chromosomal microarray (CMA) using the single-nucleotide polymorphism (SNP) array technology as the first-line investigation [7]. Management is largely symptomatic, requiring inputs from multiple specialties [5-7].

The clinicians responsible for this patient’s care felt that the degree of neurodevelopmental impairment and feeding difficulties was disproportionate for 22q11.2 deletion syndrome alone. These unexplained features prompted clinicians to consider a range of differential diagnoses, which could include Kabuki syndrome and CHARGE syndrome. Therefore, further review of the DDD study genetic results identified a pathogenic variant associated with Cornelia de Lange syndrome, 10 years after her initial 22q11.2 deletion syndrome diagnosis, clarifying the previously unexplained features [6]. 

Cornelia de Lange syndrome is a rare condition, with an estimated prevalence of one in 10,000-30,000 live births [6]. It is most commonly caused by heterozygous variants in the NIPBL gene but can also be caused by pathogenic variants in SMC1A, SMC3, RAD21, and HDAC genes [6]. Most cases arise* de novo*, although familial inheritance has been reported in an autosomal dominant or X-linked pattern, depending on the gene involved [6]. Cornelia de Lange syndrome shares some overlapping features with 22q11.2 deletion syndrome, including developmental delay, intellectual disability, behavioural difficulties, and, less frequently, feeding and gastrointestinal issues. However, it is distinguished by growth restriction, limb abnormalities, and specific distinctive facial features, most notably, synophrys (unibrow), long eyelashes, and thin, downturned lips [6-8]. As with 22q11.2 deletion syndrome, the severity of features is highly variable. Diagnosis is generally confirmed through molecular genetic testing, including gene-targeted testing and comprehensive genome testing [6]. Like 22q11.2 deletion syndrome, management is best delivered through a multidisciplinary approach, tailored to the individual’s symptoms and complications [6]. Table 1 illustrates the key features of both conditions [5-7]. 

**Table 2:** Summary comparison of 22q11.2 deletion syndrome and Cornelia de Lange syndrome. Clinical features present in the patient's case are indicated in bold. Data sources: [5-7]

	22q11.2 deletion syndrome	Cornelia de Lange syndrome	Overlapping features
Aetiology	Microdeletion at chromosome 22q11.2	Mutations in one of several genes - NIPBL, SMC1A, SMC3, HDAC8, RAD21	Genetic alteration
Common inheritance	Primarily de novo, rare familial cases	Primarily de novo, some familial cases	Primarily de novo
Prevalence	One in 4,000	One in 10,000-30,000	Rare
Common clinical features	Congenital heart defects, thymic hypoplasia leading to immunodeficiency, hypocalcaemia due to hypoparathyroidism, palatal abnormalities, growth restriction, developmental delay / intellectual disability, psychiatric disorders	Growth restriction, limb abnormalities, developmental delay, intellectual disability, feeding / gastrointestinal difficulties	Developmental delay, intellectual disability, feeding/gastrointestinal difficulties, dysmorphic features
Dysmorphic features	Broad nasal bridge, micrognathia (small chin), upward slanting palpebral fissures, low-set ears	Synophrys (unibrow), long eyelashes, thin, downturned lips, microcephaly, low-set ears	Low-set ears
Spectrum	Highly variable	Highly variable	Highly variable
Diagnosis	Molecular genetic testing - CMA	Molecular genetic testing - gene-targeted testing, comprehensive genome testing	Molecular genetic testing
Management	Multidisciplinary: Cardiac surgery, immunological support (e.g., thymus transplant, prophylactic antibiotics), calcium & vit D supplementation, development & psychiatric support	Multidisciplinary: nutritional support and feeding therapy, cardiac management, limb orthotics/surgery, developmental and behavioural therapies	Multidisciplinary support - tailored to symptoms/complications
Prognosis	Variable	Variable	Variable

To our knowledge, we present the first case of coexisting 22q11.2 deletion syndrome and Cornelia de Lange syndrome, based on a focused literature search of electronic databases.

Reports of coexisting genetic syndromes remain rare but are becoming increasingly recognised with advances in efficacy and access to genetic testing. It is estimated that 2-7% of individuals undergoing genetic testing may have more than one pathogenic variant detected [1-10]. In other dual-diagnosis cases, including those involving 22q11.2 deletion syndrome, atypical or unusually severe clinical features have also prompted re-evaluation [5]. This aligns with the evidence that a second diagnosis may only become apparent with further interpretation of existing data [2-4].

Recognising an additional genetic diagnosis can have important implications for patient care. In our case, the additional diagnosis of Cornelia de Lange syndrome allowed us to implement further targeted support, particularly with regard to feeding and developmental needs. Although the overall management plan did not change, the diagnosis provided greater clarity for both professionals and the patient’s family. It offers a scientific explanation for the severity of her symptoms and helps manage expectations around interventions, such as PEG feeding, which was documented to be a particular concern for the patient herself. Given the natural history of Cornelia de Lange syndrome, she is unlikely to maintain adequate nutritional requirements independently; the diagnosis, therefore, facilitates realistic goal-setting and open, informed discussions with the family. Cornelia de Lange syndrome is associated with complications distinct from 22q11.2 deletion syndrome, making a confirmed diagnosis valuable for guiding appropriate screening, monitoring, and follow-up. Furthermore, it enables consideration of genetic counselling and allows us to consider the potential need for parental or sibling testing.

## Conclusions

It is important to recognise that many genetic conditions have overlapping features. Once a diagnosis is made, there is a risk of anchoring bias, attributing all signs and symptoms to that condition alone. However, recent studies show that increasing numbers of individuals with developmental disorders may carry more than one pathogenic variant, highlighting that coexisting syndromes are becoming recognised more frequently. Clinicians should remain alert to features that do not fit the initial diagnosis and consider the possibility of coexisting disorders. Re-evaluation and, where appropriate, further investigation can be crucial, as identifying a second condition can help target management and better support the child’s needs.
